# Cicadas impact bird communication in a noisy tropical rainforest

**DOI:** 10.1093/beheco/arv018

**Published:** 2015-04-03

**Authors:** Patrick J. Hart, Robert Hall, William Ray, Angela Beck, James Zook

**Affiliations:** ^a^Department of Biology, University of Hawaii at Hilo, 200W. Kawili St. Hilo, HI 96720,; ^b^Department of Biology, University of Washington, 24 Kincaid Hall, Seattle WA 98105, and; ^c^Union de Ornitologos, Apdo 182–4200, Naranjo de Alajuela, Costa Rica

**Keywords:** acoustic partitioning, bird vocalizations.

## Abstract

Many animals communicate through acoustic signaling, and “acoustic space” may be viewed as a limited resource that organisms compete for. If acoustic signals overlap, the information in them is masked, so there should be selection toward strategies that reduce signal overlap. The extent to which animals are able to partition acoustic space in acoustically diverse habitats such as tropical forests is poorly known. Here, we demonstrate that a single cicada species plays a major role in the frequency and timing of acoustic communication in a neotropical wet forest bird community. Using an automated acoustic monitor, we found that cicadas vary the timing of their signals throughout the day and that the frequency range and timing of bird vocalizations closely track these signals. Birds significantly avoid temporal overlap with cicadas by reducing and often shutting down vocalizations at the onset of cicada signals that utilize the same frequency range. When birds do vocalize at the same time as cicadas, the vocalizations primarily occur at nonoverlapping frequencies with cicada signals. Our results greatly improve our understanding of the community dynamics of acoustic signaling and reveal how patterns in biotic noise shape the frequency and timing of bird vocalizations in tropical forests.

## Lay Summary

When the cicada buzz starts up, bird song shuts down in a neotropical rainforest. When birds do sing at the same time as cicadas, their song bandwidths do not overlap.

## INTRODUCTION

Acoustic signaling is the primary form of communication for many terrestrial organisms, especially birds, mammals, frogs, and insects (Rogers and Kaplan 2000; Gerhardt and Huber 2002). Acoustic space is shared by all animals in a community but can only be partitioned in 2 primary dimensions (spectral and temporal; Nelson and Marler 1990; Planque and Slabbekoorn 2008). When acoustic signals produced by different individuals overlap, interference may occur and the information in each signal may be masked (Dooling 1982; Brumm and Slabbekoorn 2005).

Because acoustic communication consumes time and energy (Prestwich 1994; Oberweger and Goller 2001), individuals should maximize the efficiency of signal transmission to receivers by reducing interference and masking from other animal signals, as well as from abiotic background noise such as wind and flowing water (Klump 1996). Interference with other animal signals and background noise may be reduced by altering the timing and/or spectral frequency of a signal, by increasing signal rate (Lengagne et al. 1999), and by increasing signal amplitude, also known as the Lombard effect (Lombard 1911; Brumm et al. 2004).

Tropical rainforests are among the most biologically and acoustically diverse places on earth (Planque and Slabbekoorn 2008) so there is great potential for competition for acoustic space in this environment. The extent to which animals are able to partition acoustic space and thus avoid signal interference in these habitats, however, is poorly known. During daylight hours, birds and insects dominate acoustic space in many neotropical rainforests, and there is evidence from these forests that some birds species do time their signals to reduce overlap with other bird species (Luther 2009). Some of the most notable sounds in neotropical forests are often produced by cicadas (Hemiptera; *Cicadidae*). Cicadas are present year-round in many tropical forests and are among the loudest calling insects known, with sound pressure levels greater than 100 dB at a distance of 50cm for some species (Sanborn and Phillips 1995). Communities of cicadas that call simultaneously have been shown to partition acoustic space (Sueur 2002), however, the effect of these cicada signals on bird communication at the community level has not been described. In this study, we address the hypothesis that birds compete with cicadas for acoustic space, and that acoustic partitioning occurs whereby birds avoid interference with cicada signals. We do this by examining the effects of the mating signal of a single cicada species on an entire community of birds in a Costa Rican rainforest. In particular, we test the prediction that there is a reduction both in the number of bird species that vocalize and in the overall rate of bird vocalizations in the forest (for all species combined) after cicadas begin signaling each day. Also, we address whether birds avoid spectral frequencies (bandwidths) used by cicadas following the onset of cicada signaling.

## METHODS

This study was conducted in primary and secondary wet forest at approximately 1100-m elevation at the Organization for Tropical Studies Las Cruces Biological field station in southern Costa Rica. We deployed an automated acoustic recorder (Songmeter SM2; Wildlife Acoustics Inc.) about 1 m above the ground in 7 different locations separated by at least 200 m. We programmed the Songmeter to record for 5min at 5-min intervals throughout the day and night from 24 June to 10 July, 2012. These months fall within the “wet” season in this forest, however, all recordings were made between 06:10 and 11:30 during sunny or partly cloudy conditions (rainfall generally began at approximately 13:00 each day) with temperatures ranging from 18 to 25 °C. Recordings were made in .WAV file format at a sampling rate of 44.1kHz using a single omnidirectional microphone (SMX-II: Wildlife Acoustics) with a sensitivity of −35 dBV/pa and frequency response of 20–20000 Hz.

*Zammara smaragdina* is a large-bodied cicada species that generally begins signaling in choruses by mid-morning. For each of 7 days at 7 different locations, we identified the time at which *Zammara* began calling and examined spectrograms of the three 5-min recording files made immediately before and after chorus onset (30min total). *Zammara* choruses generally occurred as a pulsing broadband signal throughout each of the 3 latter recordings. For all 6 recording files, we identified, tallied the number of occurrences, and measured the spectral and temporal characteristics of each bird vocalization using cursor placement in spectrograms using Raven Pro 1.4 software (Bioacoustics Research Program 2011).

Spectrograms of vocalizations were judged to be “unique” (for example, the call of the White-breasted Wood wren) based on consensus of 4 researchers (PH, RH, WR, and AB), with questionable vocalizations verified by JZ. Species identity for each vocalization was determined based on field experience by PH and especially JZ, with additional assistance from an audio CD of the bird songs of Las Cruces (Harris and Reid 2007).

All bird and cicada signals were measured using a Hann window type with a window size of 23.2ms, window overlap of 50%, and DFT (discrete Fourier transform) size of 1024 samples (Charif et al. 2010). Because each unique bird signal that was detected was recorded multiple times, and because signal characteristics can vary somewhat due to the distance of the sender from the recorder, we calculated a mean minimum and maximum frequency for each unique bird signal and for all *Zammara* choruses recorded. The difference between the mean minimum and maximum frequencies defined the *mean frequency range* for each signal. The spectral relationship (overlap) between bird and cicada signals was then categorized into 3 levels. A bird vocalization that shared 100% of its mean frequency range with the mean frequency range of a *Zammara* signal was considered a “complete” overlap; less than 100% to near zero was categorized as “partial,” and all others were categorized as “none.”

For the “partial” and “none” overlap categories, one-sample *t*-tests (R Core Development Team, version 3.0.1, 2013) were used to compare (1) the number of bird species vocalizing before versus after cicadas began signaling and (2) the total number of vocalizations before versus after cicadas began signaling. Species with vocalizations in the “complete” overlap category were not compared due to the possibility of under-counting vocalizations in this category following the onset of cicada signaling. As a control, we compared the number of vocalizations in the first three 5-min recording files to the last 3 of a 6 recording file sequence recorded on 5 mornings at 5 different locations during the study period in which *Zammara* choruses did not occur. The start times chosen for these sequences corresponded to the time of onset of *Zammara* choruses for the previous day. Little is known about the seasonality of *Zammara* choruses, however, this cicada species appears to call more regularly in the January to May dry season at Las Cruces. It is possible that this study coincided with the end of the *Zammara* chorus season, which could explain why they did not chorus each day.

We used chi-square tests in R (R Core Development Team, version 3.0.1, 2013) to determine whether the observed number of bird vocalizations that partially overlapped with cicada signals for the 3 files per day recorded just after cicadas began signaling was different than expected, based on the number of bird vocalizations that overlapped with the mean frequency range of cicada signals for the 3 files per day recorded just before cicadas began signaling.

## RESULTS

We identified 62 bird species that produced a total of 72 unique vocalizations, based on spectrogram characteristics. We were not able to assign a species name to an additional 20 unique signals, most of which were single note calls detected less than 5 times (Supplementary Appendix 1). About 17 unique signals were categorized as “complete overlap” and excluded from all subsequent analyses. The spectral bandwidth shared by birds and insects in this study was relatively narrow; 78.3% of the bird vocalizations we recorded occurred entirely within 1–8kHz. Similarly, the 95% CI for signals produced by *Zammara* cicadas ranged from a low of 2.70±0.05 to a mean high of 6.56±0.12kHz (*n* = 21 recording files). Variability in the minimum and maximum frequency of these signals was likely due primarily to distance of signaling individuals from the recorder.

### Evidence for acoustic partitioning

**Figure 1:**
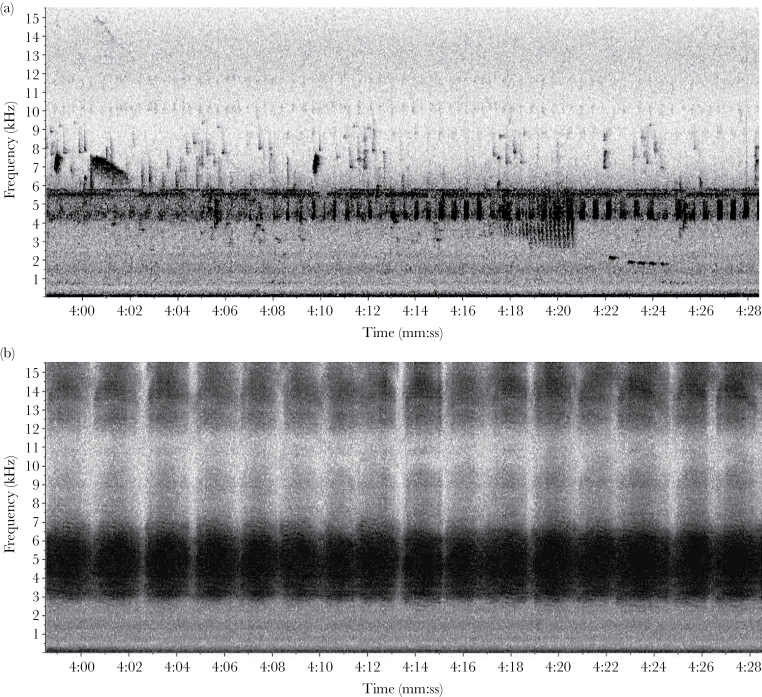
A comparison of the “soundscape” recorded during two 30 s periods from the same location on 6 July 2012, within secondary wet forest at Las Cruces Biological Station, Costa Rica. (a) A spectrogram from approximately 08:14 AM, before the onset of *Zammara* cicada choruses and shows 7 unique vocalizations (*Arremon aurantiirostris call, Picumnus olivaceus, Arremon torquatus, Catharus aurantiirostris, Arremon aurantiirostris* song, *Phaeothlypis fulvicauda, Formicarius analis*). (b) A spectrogram from approximately 08:50 AM, just after onset of *Zammara* cicada choruses, which can be seen by the dark, pulsing signal with a base frequency occupying much of the bandwidth between approximately 2.7 and 6.5 kHz. No birds are vocalizing during this period.

**Figure 2:**
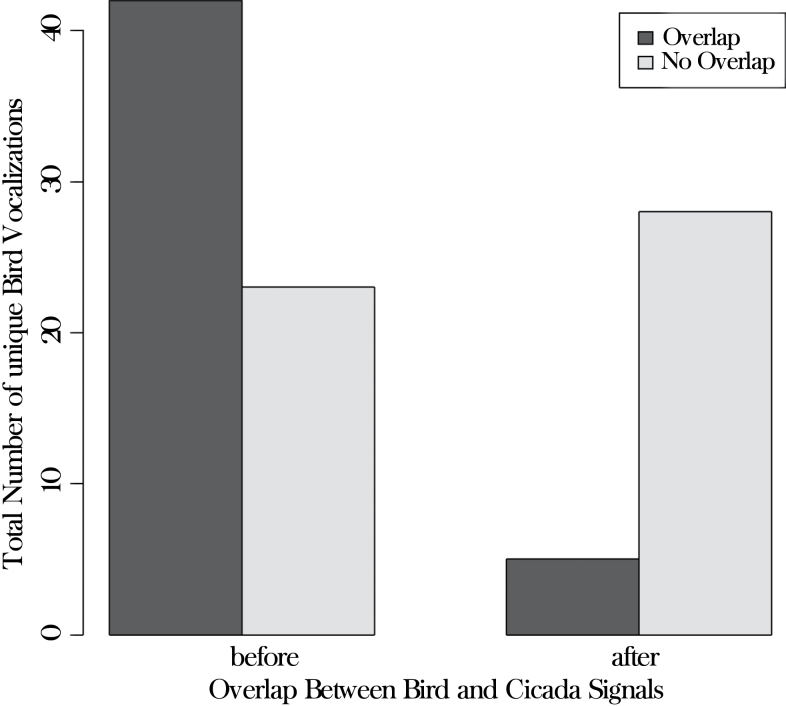
Rate of overlap, excluding “complete” overlap, between bird and cicada signals before versus after the onset of cicada signaling for 7 recording days during June and July 2012 in secondary wet forest at Las Cruces Biological Station, Costa Rica. The “overlap before” bar represents the number of unique bird vocalizations produced prior to the onset of *Zammara* chorusing, with spectra that overlap to any degree with the normal base frequency range of *Zammara* signals. The “overlap after” bar represents the number of unique bird vocalizations with spectra that overlapped to any degree with the *actual Zammara* signals.

Birds vocalized with little interference from other animal taxa for the first 2–3h after dawn. *Zammara* usually produced the first significant nonavian signals each day, with start-times ranging from 08:40 to 10:40 (Table 1). The dense broadband structure of these cicada signals would likely mask most of the more finely structured signals of birds (Figure 1a and b ; Supplementary Movie File 1a and b). However, birds in this forest appear to significantly avoid temporal overlap with cicadas by reducing and often shutting down vocalizations at the onset of cicada signal bands that utilize the same frequency range. The mean number of bird species vocalizing during a 15-min period (including unique unidentified vocalizations) immediately prior to the onset of *Zammara* signals each day was 15.7 and dropped significantly to 6.0 after the onset of *Zammara* signals (one-sample *t* = 4.01, df = 6, *P* = 0.007; Table 1). There was a similar decrease in the total number of vocalizations (for all bird species combined) produced immediately before versus after the onset of cicada signaling. The mean number of vocalizations per 15-min period dropped significantly from 435.5 to 196.1 (one-sample *T* = 6.50, df = 6, *P* = 0.0006; Table 1). For the 5 control days during which no *Zammara* signaled, there was no difference in the number of vocalizations between the first and second 15-min periods (mean = 450.6 vs. 506.0; one-sample *t* = −1.29, df = 4, *P* = 0.26). When birds did vocalize at the same time as cicadas (temporal overlap), they primarily did so at nonoverlapping frequencies. There were 42 partial overlaps and 23 no overlaps of unique bird signals with the mean cicada frequency range before cicadas began signaling, versus only 5 partial overlaps and 28 no overlaps after, a significant drop in number of overlapping vocalizations after cicadas began signaling (χ^2^ = 19.52, df = 1, *P* < 0.00001; Figure 2).

**Table 1:** The total number of bird species and vocalizations, excluding “complete” overlap, recorded in three 5-min tracks before and after the onset of cicada choruses for each day in 2012

Date	Cicada chorus start time	Number of bird species before	Number of bird species after	Total vocalizations before	Total vocalizations after
June 24	09:50	17	11	961	777
June 28	08:50	12	8	391	117
July 1	10:40	18	4	431	131
July 2	09:20	11	6	496	221
July 6	08:40	24	5	199	53
July 7	09:40	10	6	133	25
July 10	10:10	18	2	438	49

## DISCUSSION

Past work has shown that birds are able to adjust both the timing and frequency of their signals to reduce overlap with the signals of other bird species (Cody and Brown 1969; Ficken et al. 1974; Brumm and Slabbekoorn 2005; Brumm 2006). They may also adjust the frequency of signals in response to abiotic noise (Narins et al. 2004), biotic noise (Kirschel et al. 2009), and urban noise (Slabbekoorn and Peet 2003; Patricelli and Blickley 2006). This study demonstrates a significant effect of arthropod signals on communication for an entire community of birds. Cicada signaling appears to affect the number of species vocalizing, as well as the overall rate and frequency range of bird vocalizations in this forest. Most birds significantly avoided temporal overlap with cicadas by reducing and often shutting down vocalizations at the onset of cicada signals that utilize the same frequency range. Most birds also avoided spectral overlap with cicadas by vocalizing at frequencies that did not overlap with the much higher amplitude cicada signals. In general, only those species whose vocalizations do not overlap with *Zammara* continue to vocalize after the onset of *Zammara* signals.

Why do birds sing at particular frequencies and concentrate their songs at particular times of the day (often early morning)? Under the acoustic adaptation hypothesis (Morton 1975), the physical environment plays a major role in determining the most effective frequencies for sound transmission by vocalizing birds, with forest habitats favoring lower frequencies. In dense tropical wet forests, Henwood and Fabrick (1979) and Ellinger and Hödl (2003) demonstrated that physical conditions which affect sound propagation, including temperature, humidity, and wind-speed, are generally best early in the day, which at least partially explains why birds are most vocal during the morning hours. This study reveals how biotic noise in the form of cicada choruses is a factor that likely shapes the frequency and timing of bird vocalizations in tropical forests and provides an additional explanation for why birds are most vocal early in the day.

## SUPPLEMENTARY MATERIAL

Supplementary material can be found at http://www.beheco.oxfordjournals.org/

## FUNDING

Financial support was provided through an NSF LSAMP award to E. Losos (0902105) and through an NSF CREST award (0833211) to D. Price, P. Hart, E. Stacy, and M. Takabayashi.

## Supplementary Material

Supplementary Data

## References

[CIT0001] Brumm H 2006 Signalling through acoustic windows: nightingales avoid interspecific competition by short-term adjustment of song timing. J Comp Physiol A. 192(12):1279–1285. 10.1007/s00359-006-0158-x 16924503

[CIT0002] Brumm H Slabbekoorn H 2005 Acoustic communication in noise. Adv Study Beh. 35:151–209.

[CIT0003] Brumm H Voss K Köllmer I Todt D 2004 Acoustic communication in noise: regulation of call characteristics in a New World monkey. J Exp Biol. 207:443–448. 14691092 10.1242/jeb.00768

[CIT0004] Charif RA Waack AM Strickman LM 2010 Raven Pro User’s Manual. Ithaca (New York): Cornell Lab of Ornithology.

[CIT0005] Cody ML Brown JH 1969 Song asynchrony in neighbouring bird species. Nature. 222:778–780.

[CIT0006] Dooling RJ 1982 Auditory perception in birds. In: Kroodsma DE, Miller EH, editors. Acoustic communication in birds. New York: Academic Press. Vol. 1 p. 95–130.

[CIT0007] Ellinger N Hödl W 2003 Habitat acoustics of a Neotropical lowland rainforest. Bioacoustics. 13(3):297–321.

[CIT0008] Ficken RW Ficken MS Hailman JP 1974 Temporal pattern shifts to avoid acoustic interference in singing birds. Science. 183:762–763. 17790627 10.1126/science.183.4126.762

[CIT0009] Gerhardt HC Huber F 2002 Acoustic communication in insects and anurans: common problems and diverse solutions. Chicago (IL): University of Chicago Press.

[CIT0010] Harris JBC Reid L 2007 Bird Songs of Las Cruces, Costa Rica Audio CD.

[CIT0011] Henwood K Fabrick A 1979 A quantitative analysis of the dawn chorus: temporal selection for community optimization. The American Naturalist. 114(2):260–274.

[CIT0012] Kirschel AN Blumstein DT Cohen RE Buermann W Smith TB Slabbekoorn H 2009 Birdsong tuned to the environment: green hylia song varies with elevation, tree cover, and noise. Behav Ecol. 20(5):1089–1095.

[CIT0013] Klump G 1996 Bird communication in the noisy world. In: Kroodsma DE, Miller EH, editors. Ecology and evolution of acoustic communication in birds. Ithaca (NY): Cornell University Press. p. 321–338.

[CIT0014] Lengagne T Jouventin P Aubin T 1999 Finding one’s mate in a king penguin colony: efficiency of acoustic communication. Behaviour. 136(7):833–846. 10.1006/anbe.1999.1086 10373249

[CIT0015] Lombard E 1911 Le signe de l’elevation de la voix. Ann Maladies Oreille, Larynx, Nez, Pharynx. 37(101–119): 25.

[CIT0016] Luther D 2009 The influence of the acoustic community on songs of birds in a neotropical rain forest. Behav Ecol. 20(4):864–871.

[CIT0017] Morton ES 1975 Ecological sources of selection on avian sounds. Am Nat. 109(965):17–34.

[CIT0018] Narins P Feng AS Lin W Schnitzler HU Denzinger A Suthers RA Xu C 2004 Old World frog and bird vocalizations contain prominent ultrasonic harmonics. J Acoust Soc Am. 115:910–913. 15000202 10.1121/1.1636851

[CIT0019] Nelson DA Marler P 1990 The perception of birdsong and the ecological concept of signal space. In: Stebbins WC, Berkley MA, editors. Comparative Perception, Volume II: Complex Signals. John Wiley & Sons, New York.

[CIT0020] Oberweger K Goller F 2001 The metabolic cost of birdsong production. J Exp Biol. 204:3379–3388. 11606611 10.1242/jeb.204.19.3379

[CIT0021] Patricelli GL Blickley JL 2006 Avian communication in urban noise: causes and consequences of vocal adjustment. Auk. 123(3):639–649.

[CIT0022] Planque R Slabbekoorn H 2008 Spectral overlap in songs and temporal avoidance in a Peruvian bird assemblage. Ethology. 114(3):262–271.

[CIT0023] Prestwich K 1994 The energetics of acoustic signaling in anurans and insects. Am Zool. 34(6):625–643.

[CIT0024] Rogers LJ Kaplan GT 2000 Songs, roars, and rituals: Communication in birds, mammals, and other animals. USA: Harvard University Press.

[CIT0025] Sanborn AF Phillips PK 1995 Scaling of sound pressure level and body size in cicadas (Homoptera: Cicadidae; Tibicinidae). Ann Entomol Soc Am. 88(4):479–484.

[CIT0026] Slabbekoorn H Peet M 2003 Ecology: Birds sing at a higher pitch in urban noise. Nature. 424(6946):267. 12867967 10.1038/424267a

[CIT0027] Sueur J 2002 Cicada acoustic communication: potential sound partitioning in a multispecies community from Mexico (Hemiptera: Cicadomorpha: Cicadidae). Biological Journal of the Linnean Society. 75(3):379–394.

