# Supplementary material for: Cicadas impact bird communication in a noisy tropical rainforest
Source: Behav Ecol. 2015 Apr 3;26(3):839–42. doi: 10.1093/beheco/arv018 (PMC4433330; doi:10.1093/beheco/arv018)
Supplement: Supplementary Data [file supp_26_3_839__index.html]

Cicadas impact bird communication in a noisy tropical rainforest — Supplementary Data 

# Cicadas impact bird communication in a noisy tropical rainforest

## Supplementary Data

Data files

**Files in this Data Supplement:**

- Supplementary Data - Supplementary Data
- Supplementary Data - Supplementary Data
- Supplementary Data - Supplementary Data
- Supplementary Data - Supplementary Data
