# Supplementary material for: Cicadas impact bird communication in a noisy tropical rainforest
Source: Behav Ecol. 2015 Apr 3;26(3):839–42. doi: 10.1093/beheco/arv018 (PMC4433330; doi:10.1093/beheco/arv018)
Supplement: Supplementary Data [file supp_arv018_Figure_1a_b_movie_files_caption.docx]

**Figure 1a,b movie files)** A comparison of the “soundscape” recorded during two 30s periods from the same location on July 6, 2012, within secondary wet forest at Las Cruces Biological Station, Costa Rica. Figure (1a movie) is a spectrogram from approximately 08:14AM, before the onset of *Zammara* cicada choruses, and shows seven unique vocalizations (*Arremon aurantiirostris* call; *Picumnus olivaceus*; *Arremon torquatus*; *Catharus aurantiirostris*; *Arremon aurantiirostris* song; *Phaeothlypis fulvicauda*; *Formicarius analis*). Figure (1b movie) is a spectrogram from approximately 08:50AM, just after onset of *Zammara* cicada choruses, which can be seen by the dark, pulsing signal with a base frequency occupying much of the bandwidth between approximately 2.7kHz and 6.5kHz. No birds are vocalizing during this period.
