# Supplementary material for: Cicadas impact bird communication in a noisy tropical rainforest
Source: Behav Ecol. 2015 Apr 3;26(3):839–42. doi: 10.1093/beheco/arv018 (PMC4433330; doi:10.1093/beheco/arv018)
Supplement: Supplementary Data [file supp_arv018_Appendix_1.docx]

**Appendix 1**. The bird species, vocalization type, and mean frequency range for each of 92 unique vocalizations detected during the study. Before/After Cicada signifies whether the vocalization was detected only before cicadas began signaling, only after, or both.

| **Genus & Species** | **Common Name** | **Vocalization Type** | **Mean Frequency Range (kHz)** | **Before/After Cicada** |
| --- | --- | --- | --- | --- |
| *Arremon aurantiirostris* | Orange-billed Sparrow | call | 6363.51-9914.1 | Both |
| *Arremon aurantiirostris* | Orange-billed Sparrow | song | 5644.03-10291 | Both |
| *Arremon brunneinucha* | Chestnut-capped Brush Finch | song | 8608-10502 | Before |
| *Arremon brunneinucha* | Chestnut-capped Brush Finch | call | 8628-10101 | Before |
| *Arremon torquatus* | Stripe-headed Brush-Finch | song | 5302.9-8376.33 | Both |
| *Attila spadiceus* | Bright-rumped Attila | call | 2653.9-3445 | Before |
| *Automolus ochrolaemus* | Buff-throated Foliage-gleaner | call | 1428.4-2908.8 | Before |
| *Campylopterus hemileucurus* | Violet Sabrewing | song | 7116.3-9869.3 | Before |
| *Cantorchilus modestus* | Plain Wren | call | 650.5-6982.8 | Before |
| *Cantorchilus semibadius* | Riverside Wren | song | 2428.2-6134.4 | Before |
| *Catharus aurantiirostris* | Orange-billed Nightingale Thrush | song | 2746-6285.2 | Both |
| *Cercomacra tyrannina* | Dusky Antbird | song | 2493.3-3688 | Before |
| *Coereba flaveola* | Bananaquit | call | 4951.3-8472.1 | Before |
| *Colaptes rubiginosus* | Golden-olive Woodpecker | song | 2884.44-4588.06 | Before |
| *Contopus sordidulus* | Western Wood Pewee | Whistled song | 2693.9-3693.8 | Before |
| *Corapipo altera* | White-ruffed Manakin | call | 5052.6-6481.7 | Before |
| *Corapipo altera* | White-ruffed Manakin | song | 5272.25-7324 | Before |
| *Cranioleuca erythrops* | Red-faced Spinetail | song | 4856.7-6544.9 | Before |
| *Crypturellus major* | Great Tinamou | Whistled song | 1243.46-1845.08 | Both |
| *Crypturellus soui* | Little Tinamou | Whistled song | 1250-1700 | Both |
| *Dysithamnus mentalis* | Plain Antvireo | song | 986.9-1844 | Before |
| *Elaenia flavogaster* | Yellow-bellied Elaenia | song | 2311.48-3262.04 | Both |
| *Euphonia elegantissima* | Elegant Euphonia | call | 2543.9-3154.88 | Before |
| *Euphonia imitans* | Spot-crowned Euphonia | call | 2000.96-4096.25 | Before |
| *Euphonia imitans* | Spot-crowned Euphonia | song | 2541.3-5109 | Before |
| *Euphonia laniirostris* | Thick-billed Euphonia | call | 1324.6-4129.5 | Before |
| *Euphonia luteicapilla* | Yellow-crowned Euphonia | song | 2701.1-4259.4 | Before |
| *Euphonia luteicapilla* | Yellow-crowned Euphonia | call | 1506.4-2103.7 | Before |
| *Formicarius analis* | Black-faced Antthrush | song | 2209.21-3044.06 | Both |
| *Geotrygon montana* | Ruddy Quail-Dove | song | 142.9-571.65 | Both |
| *Glyphorychus spirurus* | Wedge-billed Woodcreeper | song | 4532-7804.5 | Before |
| *Habia rubica* | Red-crowned Ant Tanager | call | 1399-8535.9 | Before |
| *Habia rubica* | Red-crowned Ant Tanager | song | 2779-3350.4 | Before |
| *Heliothryx barroti* | Purple-crowned Fairy | call | 8552.2-10867.6 | After |
| *Henicorhina leucosticta* | White-breasted Wood Wren | call | 1939.4-4364.1 | Both |
| *Henicorhina leucosticta* | White-breasted Wood Wren | song | 1679.68-3894.38 | Both |
| *Hylophilus decurtatus* | Lesser Greenlet | call | 2932.4-5661.8 | Before |
| *Hylophilus decurtatus* | Lesser Greenlet | song | 1782.5-4322.6 | Before |
| *Leptotila cassini* | Gray-chested Dove | Whistled song | 311.7-610.3 | Before |
| *Lophotriccus pileatus* | Scale-crested Pygmy Tyrant | call | 2264.85-3561.33 | Both |
| *Lophotriccus pileatus* | Scale-crested Pygmy Tyrant | song | 2727-4285.3 | Before |
| *Malacoptila panamensis* | White-whiskered Puffbird | song | 5350.2-9220 | Before |
| *Megarynchus pitangua* | Boat-billed Flycatcher | call | 2167.55-4747.95 | Before |
| *Melanerpes rubricapillus* | Red-crowned Woodpecker | song | 891-4280.5 | Before |
| *Microrhopias quixensis* | Dot-winged Antwren | call | 2311.48-3262.04 | Both |
| *Milvago chimachima* | Yellow-headed Caracara | song | 4415-6298 | After |
| *Mitrephanes phaeocercus* | Tufted Flycatcher | call | 2753-3921.7 | After |
| *Momotus coeruliceps* | Blue-crowned Motmot | call | 204.2-459.4 | Before |
| *Myioborus miniatus* | Slate-throated Redstart | call | 5350.2-9116.1 | After |
| *Myiopagis viridicata* | Greenish Elaenia | song | 3610.1-5765.7 | Before |
| *Myrmeciza exsul* | Chestnut-backed Antbird | call | 5570-7792.9 | Before |
| *Myrmotherula schisticolor* | Slaty Antwren | call | 898-2428.6 | Before |
| *Odontophorus gujanensis* | Marbled Wood-Quail | call | 892-1282.3 | After |
| *Parula pitiayumi* | Tropical Parula | song | 5365.4-8719 | Before |
| *Penelope purpurascens* | Crested Guan | call | 638-944.2 | After |
| *Phaeothlypis fulvicauda* | Buff-rumped Warbler | call | 1537-2815.45 | After |
| *Phaethornis guy* | Green Hermit | call | 2000.96-9181.85 | Both |
| *Phaethornis guy* | Green Hermit | song | 5578.7-7009.1 | Both |
| *Phaethornis striigularis* | Stripe-throated Hermit | call | 5979.8-8631.2 | Both |
| *Pheugopedius rutilus* | Rufous-breasted Wren | song | 1782.5-4322.6 | Before |
| *Piaya cayana* | Squirrel Cuckoo | song | 1464.7-3939.2 | Both |
| *Picumnus olivaceus* | Olivaceous Piculet | song | 5739.8-7609.7 | Both |
| *Ramphastos swainsonii* | Chestnut-mandibled Toucan | song | 1267.5-1954.1 | Both |
| *Ramphocaenus melanurus* | Long-billed Gnatwren | song | 2675.1-3558.1 | Before |
| *Tangara gyrola* | Bay-headed Tanager | call | 6715.5-8642.5 | Before |
| *Tangara icterocephala* | Silver-throated Tanager | call | 8008-10792.2 | Before |
| *Thamnistes anabatinus* | Russet Antshrike | song | 1537.8-7883.2 | Both |
| *Thamnophilus bridgesi* | Black-hooded Antshrike | call | 892-1189.4 | After |
| *Trogon caligatus* | Gartered Trogon | song | 1236.4-1674.4 | Before |
| *Turdus assimilis* | White-throated Thrush | call | 1881.9-2727.65 | Before |
| *Xenops minutus* | Plain Xenops | song | 3454.2-6986.4 | After |
| *Zimmerius vilissimus* | Paltry Tyrannulet | song | 2500.3-3723.22 | Before |
| ud10 |  | call | 6441-7427.9 | Before |
| ud11 |  | call | 2934.8-3350.4 | Before |
| ud12 |  | Whistled song | 8622.6-10648.4 | Before |
| ud15 |  | call | 5635.9-6908.5 | After |
| ud23 |  | Whistled song | 2889.35-3467.2 | Both |
| ud3 |  | song | 4051.6-7739.6 | Before |
| ud33 |  | call | 1831-2259.5 | After |
| ud4 |  | song | 2324.45-3493.2 | Before |
| ud5 |  | song | 1584.3-2285.5 | Before |
| ud6 |  | call | 3739.9-5094.1 | Both |
| ud7 |  | call | 5298.2-6129.3 | Before |
| ud8 |  | song | 2570-5660 | Before |
| ud9 |  | call | 5791.7-7765.5 | Before |
| udDD |  | call | 5153.5-6409.9 | Before |
| udEE |  | call | 2942.9-8555.9 | Before |
| udGG |  | song | 6480.5-10409.7 | Before |
| udH |  | call | 6609.2-7527.9 | Before |
| udHH |  | call | 7815.3-8049.2 | Before |
| udK |  | song | 6507.2-9467.3 | Before |
| udL |  | call | 5435.5-6175.4 | Before |
